# Two-Stage Fast DOA Estimation Based on Directional Antennas in Conformal Uniform Circular Array

**DOI:** 10.3390/s21010276

**Published:** 2021-01-03

**Authors:** Yao Xie, Mo Huang, Yuanyuan Zhang, Tao Duan, Changyuan Wang

**Affiliations:** 1 Institute of Microelectronics, Chinese Academy of Sciences, Beijing 100029, China; xieyao@ime.ac.cn (Y.X.); huangmo@ime.ac.cn (M.H.); zhangyuanyuan@ime.ac.cn (Y.Z.); duantao@ime.ac.cn (T.D.); 2 University of Chinese Academy of Sciences, Beijing 100049, China

**Keywords:** direction-of-arrival (DOA), directional antenna, conformal UCA, two-stage, decouple, multi-target

## Abstract

In conformal array radar, due to the directivity of antennas, the responses of the echo signals between different antennas are distinct, and some antennas cannot even receive the target echo signal. These phenomena significantly affect the accuracy of direction-of-arrival (DOA) estimation. To implement accurate DOA estimation in a conformal uniform circular array (UCA) composed of directional antennas, the two-stage fast DOA estimation algorithm is proposed. In the pre-processing stage, multi-target decoupling and target detection are mainly used to obtain the targets’ range bin indexes set; in the rough-precise DOA estimation stage, the amplitude and phase information of each antenna are used for rough DOA estimation and precise DOA estimation, respectively. Based on simulation and actual anechoic chamber radar experiments, and through quantitative analyses of the accuracy, validity and elapsed time of the two-stage fast DOA estimation algorithm compared with the directional antenna MUSIC (DA-MUSIC), sub-array MUSIC (S-MUSIC) and Capon-like algorithms, results indicate that the two-stage fast DOA estimation algorithm can rapidly and accurately estimate DOAs in a multi-target scenario without the range-angle pair-matching procedure. Lower computational complexity and superior estimation accuracy provide the two-stage fast DOA estimation algorithm a broader application prospect in the practical engineering field.

## 1. Introduction

Direction-of-arrival (DOA) estimation has received considerable attention in the field of radar, sonar and mobile communications [1,2,3], because of its important role in the array signal processing field for locating targets from their echo signal [4]. Compared with a traditional array, the conformal array is a smooth distribution array with better aerodynamics and a more compact form factor [5,6,7]. Accurate DOA estimation will contribute to the application of the conformal array in airborne radar, ship-based radar and missile-borne radar [8].

After decades of development, a large number of DOA estimation algorithms have been proposed with excellent performance [9,10,11], such as the maximum likelihood (ML) estimation algorithm, based on the idea of maximum likelihood [12], the Capon beam forming algorithm based on beam forming [13], multi signal classification (MUSIC) [14] and estimating signal parameter via rotational invariance techniques (ESPRIT) [15], which use algorithms based on subspace decomposition, the spare signal reconstruction, and compression-based algorithms that use compressed sensing [16,17], etc. These algorithms can estimate DOA accurately in the majority of scenarios. However, all of these proposed algorithms are based on omnidirectional antennas. In other words, all elements in the array have no directivity to the target response in all directions, and the influence of antenna directivity on DOA estimation performance need not be considered. In a conformal array, because of the shadow effect of the metal carrier [18], omnidirectional antennas cannot be realized, so most of the antennas arranged in conformal arrays are directional antennas. For directional arrays, due to the different response intensities of the target echo on different antennas, even some antennas cannot receive target echo signal [19,20], thus there is a shadow effect. Based on the above problems, DOA estimation algorithms based on omnidirectional antennas cannot be directly transplanted to conformal arrays.

Some effective DOA estimation algorithms are proposed based on the properties of directional antennas. For the directional antenna MUSIC (DA-MUSIC) algorithm [21], first of all, the antenna pattern is used to modify the steering vector, and then, the MUSIC algorithm is used to conduct DOA estimation on the directional antenna. Therefore, the DA-MUSIC algorithm requires an accurate antenna pattern function as prior knowledge. Similarly, the Capon-like algorithm [22] also uses the antennas pattern to correct the steering vector, so as to employ Capon beam-forming algorithm on directional antennas. Unfortunately, in actual radar, the accurate antenna pattern function is difficult to obtain. In order to avoid the requirement of prior knowledge of antenna pattern information, the array interpolation DOA estimation algorithm transforms an array composed of directional antennas into an ideal array composed of omnidirectional antennas through interpolation [23,24], and the constructed ideal array structure needs to be similar to the actual array structure [25]. Even so, the interpolation error will also affect the final DOA estimation accuracy. The sub-array MUSIC (S-MUSIC) algorithm divides the array into multiple sub-arrays to ensure that at least one sub-array corresponding to the array steering vector is full rank [26], and then DOA estimation is performed on each sub-array using the MUSIC algorithm. The S-MUSIC algorithm does not need the prior knowledge of the antennas pattern and does not perform interpolation processing, so there is no interpolation error. However, the S-MUSIC algorithm reduces the array aperture [27], and the spatial spectrum obtained is discontinuous, so the estimation performance is poor.

In order to realize accurate estimation of multi-target DOAs on a conformal uniform circular array (UCA) composed of directional antennas, a two-stage fast DOA estimation algorithm is proposed to solve the problem that directional antennas respond differently to the target amplitude. The two-stage fast DOA estimation algorithm consists of pre-processing and a rough-precise DOA estimation stage: (1) In the pre-processing stage, multi-target decoupling and target detection are mainly used to obtain the set of target range bin indexes. Through discrete Fourier transform (DFT) processing, several information targets located at different ranges are decoupled; for each antenna, the “M/N criterion” [28] is used to obtain the target range bin index along the array dimension after target detection to overcome the influence of different target amplitude responses brought by the directional antennas on the detection performance. (2) In the stage of rough-precise DOA estimation, the amplitude and phase information of each antenna are used for rough DOA estimation and precise DOA estimation, respectively. Different sub-arrays are divided according to the beam coverage area, and the target DOA rough estimation is realized by the amplitude differences of different sub-arrays; based on the amplitude approximation of a single sub-array and the phase information of the target on the antenna, the MUSIC algorithm is used to search the spectrum peak in its field of view (FOV) to realize the DOA’s precise estimation of the target.

In general, the main contributions of this paper are listed below:

- Firstly, the two-stage fast DOA estimation algorithm decouples each target from the range dimension through DFT processing in the pre-processing stage, so that there is no need for a subsequent range-angle pair-matching procedure.
- Secondly, the proposed algorithm required little prior knowledge of the antennas’ patterns in the precise DOA estimation stage, and only needs a beam width of 3 dB; in other words, there is no need for the precise antenna pattern.
- Thirdly, the proposed algorithm selects a single sub-array and search spectrum peaks in its FOV and estimates the DOA precisely in the rough-precise DOA estimation stage, which reduces the computational burden.

The rest of this paper is organized as follows: In Section 2, the transmitted linear frequency modulate (LFM) signal, modal in conformal UCA and composed of directional antennas is introduced. In Section 3, the specific steps of the two-stage fast DOA estimation algorithm is introduced, along with its computational complexity. In Section 4, simulation and anechoic chamber experiments are carried out, respectively, and the results are compared and analyzed. Finally, the conclusions of this study are summarized in Section 5.

## 2. Signal Model

**Figure 1:**
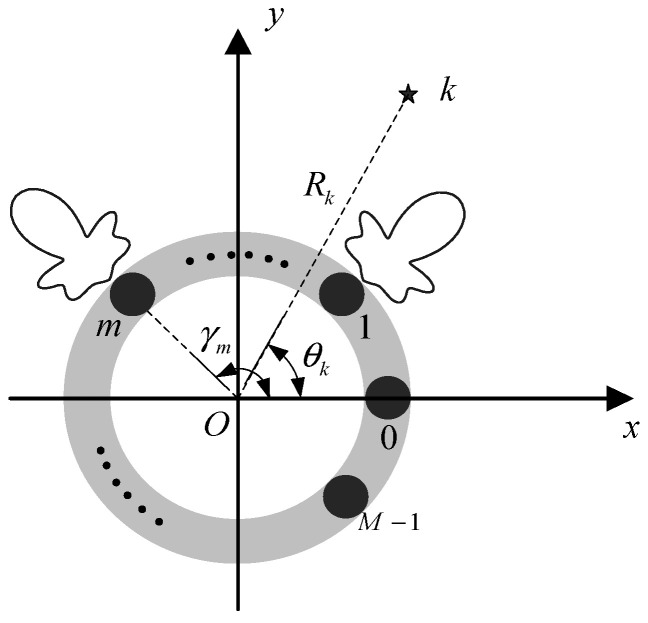
Radar array geometry diagram.

Assume that a UCA radar is composed of M directional antennas; its array geometry is shown in Figure 1. For the mth antenna *(*0≤m≤M−1), the distance from the origin of coordinate is r and the positive angle with the *x*-axis is $\gamma_{m}$. Suppose that there are K(K<M) uncorrelated far-field targets in multi-target scenarios and the position of the kth target on the polar coordinate plane is expressed as $\left\{R_{k},\theta_{k}\right\}$ (1≤k≤K), where $R_{k}$ and $\theta_{k}$ denote the distance from origin of coordinate to the target and angle measured from *x*-axis, respectively.

For a UCA radar, the assumption is that a reference antenna is at the origin of the coordinate system, and the power of the transmitted signal and the echo signal, without the weighting of the antenna pattern, are set as constant 1. Under this assumption, in a single transmitting pulse period, the transmitted LFM signal $s_{o}(t)$ for the reference antenna is:

(1) $$s_{o}(t)=\exp(j2\pi (f_{0}t+\frac{1}{2}\mu t^{2}))\;t\in (0,T_{p})$$

where $f_{0}$ is the start frequency for the transmitted signal and λ is corresponding signal wavelength. $T_{p}$ and μ are the pulse width and the slope of the frequency modulation for the transmitted signal, respectively. The transmitted signal reflected by the targets and received by the antennas, and the delay time between the transmitted signal and the received signal for the kth target is $\tau_{k}=\frac{2R_{k}}{c}$. The received echo signal of the kth target by the reference antenna is:

(2) $$s_{r}(t)=\exp(j2\pi (f_{0}(t-\tau_{k})+\frac{1}{2}\mu {\left(t-\tau_{k}\right)}^{2}))$$

For the kth target, after de-chirp processing in the analog domain and low-pass filter processing, the corresponding beat signal $s_{k}\left(t\right)$ is:

(3) $$s_{k}(t)=\exp(j2\pi f_{0}\tau_{k}-j\pi D\tau_{k}^{2}+j2\pi \mu \tau_{k}t)$$

The beat signal $s_{k}\left(t\right)$ in the analog domain is converted to the digital domain by sampling; the corresponding beat signal $s_{k}\left(l\right)$ in the digital domain is:

(4) $$s_{k}(l)=\exp(j2\pi f_{0}\tau_{k}-j\pi \mu \tau_{k}^{2}+j2\pi \mu \tau_{k}T_{s}l)\;l=0,\;1,\;\cdots ,\;L-1,$$

where L denotes the number of snapshots, and $T_{s}$ denotes the sampling interval. For multi-target scenarios, the corresponding beat signal vector of multiple targets is $S\left(l\right)=\left[s_{1}\left(l\right)\;s_{2}\left(l\right)\;\cdots \;s_{K}\left(l\right)\right]\in {\mathbb{C}}^{M\times 1}$. For the kth target, the wavefront arrival time difference between the reference antenna and the mth antenna is $\chi_{m,k}$, and the corresponding phase difference is $\omega_{m,k}=2\pi f_{0}\chi_{m,k}$.

(5) $$\chi_{m,k}=\frac{r\cos(\gamma_{m}-\theta_{k})}{c}=\frac{r\cos(\frac{2m\pi }{M}-\theta_{k})}{c}$$

For directional antennas, the power of the received signal for each antenna is weighted by the antenna pattern. Then, the modified steering vector $a_{k}\in {\mathbb{C}}^{M\times 1}$ for kth target is formulated as

(6) $$a_{k}={\left[\begin{array}{cccc} g_{0}(\theta_{k})\exp(j\omega_{0,k}) & g_{1}(\theta_{k})\exp(j\omega_{1,k}) & \cdots & g_{2}(\theta_{k})\exp(j\omega_{M-1,k}) \end{array}\right]}^{T},$$

where ${\left(\cdot \right)}^{T}$ denotes a transpose operation. And $g_{m}(\theta_{k})$ is the amplitude weighting term of the antenna pattern of the mth antenna to the kth target.

In multi-target scenarios, the steering matrix is $A\left(\theta \right)=\left[a_{1}\;a_{2}\;\cdots \;a_{K}\right]\in {\mathbb{C}}^{M\times K}$. Therefore, the corresponding array output data $X\left(l\right)\in {\mathbb{C}}^{M\times 1}$ in the array radar is [29]:

(7) X(l)=A(θ)S(l)+N(l),

where $N\left(l\right)\in {\mathbb{C}}^{M\times 1}$ is additive white Gaussian noise (AWGN). For LFM array radar, the range and angle information are contained in the array output data X(l), which can be used as the basic data for the next steps of the target detection and DOA estimation.

## 3. Two-Stage Fast DOA Estimation Algorithm

**Figure 2:**
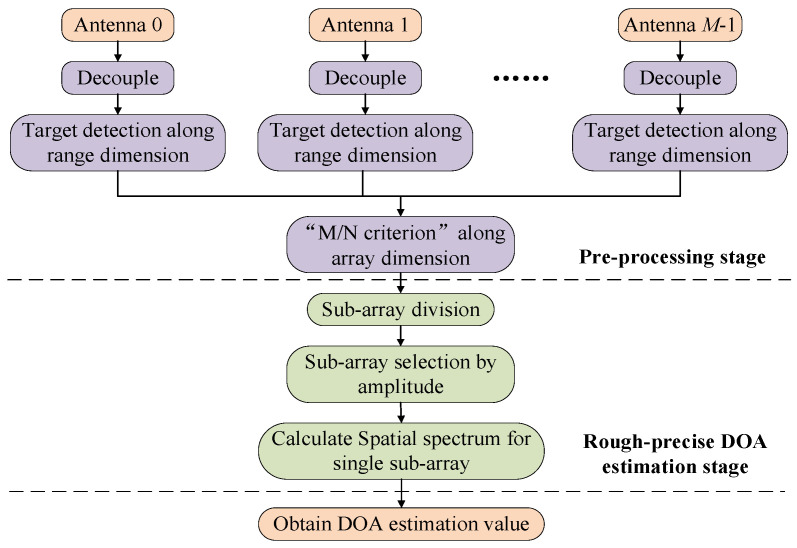
Two-stage fast DOA estimation algorithm flow chart.

In this section, a two-stage fast DOA estimation algorithm is proposed for the UCA composed of directional antennas, and the flow chart of the proposed algorithm is shown in Figure 2. Firstly, the specific steps in the pre-processing stage are introduced, including multi-target information decoupling and target detection. Then, the detailed algorithm of the DOA estimation stage is introduced, which includes a rough DOA estimation and a precise DOA estimation by using the amplitude and phase information of the target on each antenna. Finally, the computational complexity of two-stage fast DOA estimation algorithm is analyzed.

### 3.1. Pre-Processing Stage

The main steps in the pre-processing stage are multi-target information decoupling and target detection along the range dimension. First of all, in order to realize the decoupling of multi-target information, discrete Fourier transform (DFT) processing is carried out along the fast-time dimension for each antenna. Through DFT processing, the frequency spectrum $F_{m}(i)$ of the mth antenna is shown as follows:

(8) $$\begin{array}{l} F_{m}(i)=\sum_{l=0}^{L-1}X[m,l]\exp(-j2\pi il/L)\\ \;\;\;\;=\sum_{l=0}^{L-1}(\sum_{k=1}^{K}g_{m}(\theta_{k})\exp(j\omega_{m,k})\exp(j2\pi f_{0}\tau_{k}-j\pi \mu \tau_{k}^{2}+j2\pi \mu \tau_{k}T_{s}l))\exp(-j2\pi il/L)\\ \;\;\;\;=\sum_{k=1}^{K}(g_{m}(\theta_{k})\exp(j\omega_{m,k})\exp(j2\pi f_{0}\tau_{k}-j\pi \mu \tau_{k}^{2})\cdot \sum_{l=0}^{L-1}\exp(j2\pi \mu \tau_{k}T_{s}l-j2\pi il/L)) \end{array}\;,$$

where X[m,l] represents the array output data, *m* and *l* are the row and column elements, respectively. However, for LFM array radar, the beat signal after de-chirping is the superposition of multiple single-frequency signals, and the target range is proportional to the signal frequency. DFT not only implements time-frequency domain conversion of the echo signal [30], but also implements the conversion of the fast-time sampling and range data.

For the kth target, when μτkTs=IkL, it is known from Equation (8) that the phase of the beat signal is correlated with the twiddle factor of the DFT. Therefore, the range dimension spectrum $F_{m}\left(i\right)$, after the superposition of sampling data is:

(9) $$F_{m}(i)=L\cdot \sum_{k=1}^{K}\left(g_{m}(\theta_{k})\exp(j\omega_{m,k}\right)\exp(j2\pi f_{0}\tau_{k}-j\pi \mu \tau_{k}^{2}))\delta (i-I_{k}\;)\;i=0,\;1,\;\cdots ,\;L-1,$$

where δ(i) denotes the Dirac function. Finally, the amplitude and phase information of the multi-target are compressed into a single range bin through DFT to realize the information decoupling of different range targets [31].

The second step in the pre-processing stage is target detection. In order to remove the influence of the weighting effect of the antenna pattern and the shadow effect of the array carrier on the detection performance, firstly, the CFAR-like detection algorithm [32] is used to perform detection of targets along the range dimension of each antenna; then, for the obtained antenna-range coding matrix $I^{c}\in R^{M\times L}$, the element $I^{c}\left[m,l\right]$ in the matrix is:

(10) $$I^{c}\left[m,l\right]=\left\{ \begin{array}{l} 1\;|F_{m}(l)\geq \eta_{m,l}|\\ 0\;|F_{m}(l)<\eta_{m,l}| \end{array}\right.,$$

where |·| denotes the modulo operation, and $\eta_{m,l}$ denotes the detection threshold corresponding to the CFAR-like algorithm on the lth range bin of the mth antenna. On this basis, for all antennas, the “M/N” criterion is used to detect each range bin along the array dimension, and the range bin indexes set obtained after detection is $I=\{I_{k}|k=1,2,\cdots ,K\}$, where $I_{k}$ denotes the range bin index corresponding to the kth target. In the conformal array composed of directional antennas, through the CFAR-like detection algorithm and the “M/N” criterion, it is possible to avoid the missed alarm caused by the failure of detecting the target caused by the use of CFAR-like detection algorithm under a single antenna. At the same time, false alarms caused by strong interference noise can also be avoided.

### 3.2. Rough-Precise DOA Estimation Stage

For kth target, ignoring the amplitude term L for Equation (9), the corresponding array dimension vector $F(I_{k})$ is:

(11) $$\begin{array}{c} F(I_{k})={\left[\begin{array}{cccc} F_{0}(I_{k}) & F_{1}(I_{k}) & \cdots & F_{M-1}(I_{k}) \end{array}\right]}^{T}\\ \;\;\;\;={\left[\begin{array}{cccc} g_{0}(\theta_{k})\exp(j\omega_{0,k}+j\Psi_{k}) & g_{1}(I_{k})\exp(j\omega_{1,k}+j\Psi_{k}) & \cdots & g_{M-1}(I_{k})\exp(j\omega_{M-1,k}+j\Psi_{k}) \end{array}\right]}^{T}\\ \;\;\;\;={\left[\begin{array}{cccc} g_{0}(\theta_{k}) & g_{1}(\theta_{k}) & \cdots & g_{M-1}(\theta_{k}) \end{array}\right]}^{T}⊙{\left[\begin{array}{cccc} \exp(j\omega_{0,k}+j\Psi_{k}) & \exp(j\omega_{1,k}+j\Psi_{k}) & \cdots & \exp(j\omega_{M-1,k}+j\Psi_{k}) \end{array}\right]}^{T}\\ \;\;\;\;=g_{k}⊙\varphi_{k} \end{array}$$

where (⊙) denotes Hadamard product. Equation (11) shows not only the amplitude information, but also the phase information contained in $F\left(I_{k}\right)\;$[33]. In the directional antennas, the amplitude dimension information can be used for DOA estimation due to the difference of the amplitude term $g_{k}$ of each antenna caused by the antenna pattern. Then, rough-precise DOA estimation based on amplitude and phase information is realized.

Amplitude information is utilized to implement rough DOA estimation. For a UCA, the angle interval between two adjacent antennas is 2πM, the 3 dB beam width of each antenna in azimuth is $\theta_{0.5}$ for an antenna, and the number of antennas covered by the 3 dB beam is:

(12) $$M_{p}=\left\lfloor \frac{M\theta_{0.5}}{2\pi }\right\rfloor$$

where ⌊·⌋ is the floor function.

In order to solve the rank defect problem of the steering vector in UCA radar, the array is divided into several sub-arrays, and an antenna’s number in a sub-array is $M_{p}$. Compared with the traditional sub-array division estimation algorithm, the two-stage fast DOA estimation algorithm increased the steps of sub-array selection processing. There are two obvious advantages to increasing this step. First, the computational burden is lower because it only needs to search spectrum peaks in single sub-array FOVs instead of calculating the spatial spectrum of all angles. Second, the interference caused by other sub-arrays for search spectrum peaks is avoided. For the proposed algorithm, the sub-array division and sub-array selection are realized by the constructed sub-array division matrix $A_{s}$, then the corresponding sub-arrays amplitudes matrix $G_{s,k}$ is shown as follows:

(13) $$\begin{array}{c} G_{S,k}{=A}_{S}\cdot \xi_{S,k}={\left[\begin{array}{cccccc} 1 & \cdots & 1 & 0 & \cdots & 0\\ 0 & 1 & \cdots & 1 & 0 & \cdots \\ \cdots & 0 & 1 & \cdots & 1 & 0\\ & & \vdots & \vdots & & \\ \cdots & 1 & 0 & \cdots & 0 & 1 \end{array}\right]}_{M\times M}\cdot {\left[\begin{array}{c} g_{0}(\theta_{k})\\ g_{1}(\theta_{k})\\ \vdots \\ g_{M-1}(\theta_{k}) \end{array}\right]}_{M\times 1}\\ ={\left[\begin{array}{c} g_{0}(\theta_{k})+g_{1}(\theta_{k})+\cdots +g_{M_{p}-1}(\theta_{k})\\ g_{1}(\theta_{k})+g_{2}(\theta_{k})+\cdots +g_{M_{p}}(\theta_{k})\\ \vdots \\ g_{M-1}(\theta_{k})+g_{0}(\theta_{k})+\cdots +g_{M_{p}-2}(\theta_{k}) \end{array}\right]}_{M\times 1} \end{array}.$$

For consecutive $M_{p}$ antennas, if the target falls in the FOV corresponding to the sub-array, then the target amplitudes weighted by the antenna pattern will be stronger than the other sub-arrays. In this case, the rough DOA estimation can be realized by comparing the strength of each element in $G_{s,k}$. If the location index of the strongest element in $G_{s,k}$ is q, that is, the kth target is located in the FOV of the qth sub-array. Correspondingly, the estimation precision of rough estimation is $\frac{1}{2}M_{p}\theta_{0.5}$, which is related to the size of the sub-array FOV.

Based on the result of the rough DOA estimation, the precise DOA estimation is carried out by using phase information. For the qth sub-array, its antennas location indexes vector $I^{q}$ is:

(14) $$I^{q}={\left[\begin{array}{cccc} q\backslash M & q+1\backslash M & \cdots & q+M_{p}-1\backslash M \end{array}\right]}^{T}$$

where (\) denotes remainder operation; then the sub-array dimension vector $F^{q}\left(I_{k}\right)$ is:

(15) $$F^{q}(I_{k})={\left[\begin{array}{cccc} F_{I^{q}[1]}(I_{k}) & F_{I^{q}[2]}(I_{k}) & \cdots & F_{I^{q}[M_{p}]}(I_{k}) \end{array}\right]}^{T}=g_{k}^{q}⊙\varphi_{k}^{q}.$$

The corresponding amplitude term $g_{k}^{q}$ and phase term $\varphi_{k}^{q}$ are shown as following, respectively.

(16) $$\{\begin{array}{l} g_{k}^{q}={\left[\begin{array}{cccc} g_{I^{q}[1]}(\theta_{k}) & g_{I^{q}[2]}(\theta_{k}) & \cdots & g_{I^{q}[M_{p}]}(\theta_{k}) \end{array}\right]}^{T}\\ \varphi_{k}^{q}=\exp(j\Psi_{k})\cdot {\left[\begin{array}{cccc} \exp\left(j\omega_{{}_{I^{q}[1]},k}\right) & \exp\left(j\omega_{{}_{I^{q}[2]},k}\right) & \cdots & \exp\left(j\omega_{{}_{I^{q}[M_{p}]},k}\right) \end{array}\right]}^{T} \end{array}.$$

Because $\frac{2\pi M_{p}}{M}\leq \theta_{0.5}$, the amplitude term $g_{k}^{q}$ can be approximated:

(17) $$g_{I^{q}[1]}(\theta_{k})\approx g_{I^{q}[2]}(\theta_{k})\approx \cdots \approx g_{I^{q}[M_{p}]}(\theta_{k}).$$

For array radars, the array amplitude error and phase error will reduce the DOA estimation performance [34]. However, the amplitude error only affects the intensity of spatial spectrum peaks, and does not affect its position, so the DOA estimation value after amplitude approximation is still accurate. After amplitude approximation, the sub-array dimension vector $F^{q}\left(I_{k}\right)$ for the qth sub-array is:

(18) $$\begin{array}{cc} F^{q}(I_{k}) & ={\left[\begin{array}{cccc} g_{I^{q}[1]}(\theta_{k}) & g_{I^{q}[2]}(\theta_{k}) & \cdots & g_{I^{q}[M_{p}]}(\theta_{k}) \end{array}\right]}^{T}⊙\varphi \\ & =\exp(j\Psi_{k})\underset{\mathrm{Phase}\;\mathrm{information}\;\mathrm{for}\;\mathrm{DOA}}{\underset{︸}{\left[\begin{array}{cccc} \exp(j\omega_{I^{q}[1],k}) & \exp(j\omega_{I^{q}[2],k}) & \cdots & \exp(j\omega_{I^{q}[M_{p}],k}) \end{array}\right]}} \end{array}.$$

It can be seen from Equation (18) that the amplitude of each element in $F^{q}\left(I_{k}\right)$ is same, and at this time, the phase difference between different antennas is the basis of precise DOA estimation.

When there are no errors in the array, MUSIC can be directly used to achieve the precise DOA estimation for the qth sub-array. At this time, the sub-array dimension vector of the qth sub-array is $F^{q}\left(I_{k}\right)$ and the corresponding covariance matrix for $F^{q}\left(I_{k}\right)$ is $R_{xx}$. Eigen-decomposition (EVD) of $R_{xx}$ can be obtained as follows:

(19) $$R_{xx}=E[F^{q}(I_{k})\cdot {\left(F^{q}(I_{k})\right)}^{H}]=U_{k}^{N}\Lambda_{k}^{N}{\left(U_{k}^{N}\right)}^{H}+U_{k}^{S}\Lambda_{k}^{S}{\left(U_{k}^{S}\right)}^{H},$$

where ${\left(\cdot \right)}^{H}$ denotes the conjugate transpose operation. $U_{k}^{N}$ and $U_{k}^{S}$ denote the noise subspace and signal subspace for the kth target, respectively. For the qth sub-array, the steering matrix $A_{\theta }^{q}$ is:

(20) $$A_{\theta }^{q}=\left[\begin{array}{ccc} \begin{array}{c} \exp(j2\pi r\cos(\gamma_{I^{q}[1]}-\theta_{1})/\lambda )\\ \exp(j2\pi r\cos(\gamma_{I^{q}[2]}-\theta_{1})/\lambda )\\ \vdots \\ \exp(j2\pi r\cos(\gamma_{I^{q}[M_{p}]}-\theta_{1})/\lambda ) \end{array} & \begin{array}{c} \cdots \\ \cdots \\ \ddots \\ \cdots \end{array} & \begin{array}{c} \exp(j2\pi r\cos(\gamma_{I^{q}[1]}-\theta_{K})/\lambda )\\ \exp(j2\pi r\cos(\gamma_{I^{q}[2]}-\theta_{K})/\lambda )\\ \vdots \\ \exp(j2\pi r\cos(\gamma_{I^{q}[M_{p}]}-\theta_{K})/\gamma ) \end{array} \end{array}\right].$$

In the FOV of the qth sub-array, the corresponding spatial spectrum P(θ) is:

(21) $$P(\theta )=\frac{1}{a^{q}{\left(\theta \right)}^{H}U_{k}^{N}{\left(U_{k}^{N}\right)}^{H}a^{q}(\theta )}\;\;\theta \in [\gamma_{I^{q}[1]},\frac{2\pi (M_{p}-1)}{M}+\gamma_{I^{q}[1]}].$$

Precise DOA estimation is realized by spectrum peak-searching for the spatial spectrum of a single sub-array FOV, with the estimation precision of precise DOA estimation is $\frac{\kappa }{2}$, where κ denotes the peak-searching angle interval.

In summary, the main steps of the two-stage fast DOA estimation algorithm can be described as follows Algorithm 1.
**Algorithm 1** Two-stage fast DOA estimation algorithm
**Input:** Array output data *X***Output:** Targets DOA estimation values ${\hat{\theta }}_{1},\;{\hat{\theta }}_{2},\;\cdots ,\;{\hat{\theta }}_{k}$
**Pre-processing stage;**1:**Step 1:** According to Equation (8), decouple the information of multi-target to realize the conversion between the fast-time sampling and range dimension.2:**Step 2:** Detect target along range dimension for each antenna and use “M/N criterion” to obtain the range indexes set $I=\{I_{k}|k=1,2,\cdots ,K\}$.
**Rough-precise DOA estimation stage;**3:**for**k=1,2,⋯,K**do**4: **Step 3:** For kth target, according to Equation (11), obtain the array dimension vector $F(I_{k})$ in same range index $I_{k}$.5: **Step 4:** For array dimension vector $F(I_{k})$, the sub-array division and the selection of the one with the maximum value carried out to realize the rough DOA estimation and obtain the sub-array dimension vector $F^{q}(I_{k})$.6: **Step 5:** Calculate the $R_{\mathrm{xx}}$ for $F^{q}(I_{k})$ and perform EVD for $R_{\mathrm{xx}}$. Based on Equation (21), calculate the spatial spectrum of kth target. Complete precise DOA estimation ${\hat{\theta }}_{k}$ by spectrum peak-searching.7:**end for**

### 3.3. Computational Complexity

The computational complexity of the two-stage fast DOA estimation algorithm is evaluated by calculating the number of complex multiplication operations [35] among different algorithms (DA-MUSIC, S-MUSIC, Capon-like, see Table 1). In the following analysis, the $N_{s}$ denotes the number of sub-arrays in the S-MUSIC algorithm and $M_{s}$ denotes the number of antennas in a single sub-array in the S-MUSIC algorithm. The computational complexity of the proposed two-stage fast DOA estimation algorithm is dominated by three parts: (1) calculate the covariance matrix $R_{xx}$ for the sub-array dimension vector, (2) EVD for $R_{xx}$, and (3) 1D spectrum peak-searching in FOV of a single sub-array. The computational complexity of calculating $R_{xx}$ is $O\left({M_{q}}^{2}\right)$ and the complexity of calculating EVD $R_{xx}$ is $O\left({M_{q}}^{3}\right)$ [36]; then the complexity of spectrum peak-searching is $O\left(\frac{2\pi \left(M_{q}-1\right)}{M\kappa }K\left(2M_{q}\left(M_{q}-1\right)+4\left(M_{q}-1\right)\right)\right)$.

It can be seen from Table 1: First, since $M_{q}<M$ and L≫1 at the same time, the computational complexity of the proposed two-stage fast DOA estimation algorithm in calculating the covariance matrix $R_{xx}$ is lower than that of the other three algorithms. Secondly, since $M>M_{q}$, the computational complexity of the two-stage fast DOA estimation algorithm when EVD is performed on $R_{xx}$ is lower than that of DA-MUSIC and Capon-like algorithms. As for the S-MUSIC algorithm, it also needs to be divided into sub-arrays; at the same time $N_{s}>1$, then compared to the S-MUSIC algorithm, the two-stage fast DOA estimation algorithm has approximate or lower computational complexity when performing EVD on $R_{xx}$. Finally, in actual radar work M≥K, $\frac{K}{M}<1$, $\left(M-K)>(M_{q}-1\right)$ is usually satisfied, so the two-stage fast DOA estimation algorithm will have the lowest computational complexity when searching for spectral peaks. Through analyzing the computational complexity of the three main parts, it can be found that the two-stage fast DOA estimation has the lowest computational burden.

Consider a numerical scenario where the antenna number M=8, the number of snapshots L=1000, the number of targets K=3 and the angle interval κ=0.1. In addition, the number of sub-arrays in the S-MUSIC algorithm $N_{s}=4$, and the number of antennas in a single sub-array in the S-MUSIC algorithm $M_{s}=3$, which is same with $M_{q}$, respectively. In this scenario, the three parts ${M_{q}}^{2}$, ${M_{q}}^{3}$, $\frac{2\pi \left(M_{q}-1\right)}{M\kappa }K\left(2M_{q}\left(M_{q}-1\right)+4\left(M_{q}-1\right)\right)$ of two-stage fast DOA estimation are equal to 9, 27 and 27,000 respectively, and the sum of these three parts is 27,036. As for other three comparison algorithms, the computational complexities are 453,312, 90,216 and 525,320, respectively. Through the numerical analysis, the excellent performance of two-stage fast DOA estimation algorithm is shown clearly.

## 4. Simulations and Anechoic Chamber Experiments

**Figure 3:**
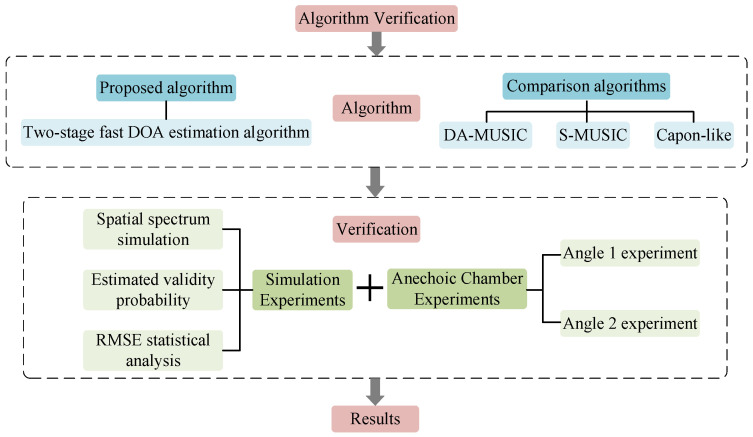
Algorithm verification flowchart.

The performance of the two-stage fast DOA estimation algorithm is verified by simulation and anechoic chamber experiments, and the specific experimental process is shown in Figure 3. In simulation experiments, the following are calculated: statistical analysis of the spatial spectrum, estimation accuracy and RMSE results between DA-MUSIC, S-MUSIC, Capon-like and the two-stage fast DOA estimation algorithms. In the anechoic chamber experiments, the performance of different algorithms (DA-MUSIC, S-MUSIC, Capon-like and two-stage fast DOA estimation) in different angle scenarios was verified using the actual conformal UCA radar. The superiority of the two-stage fast DOA estimation algorithm over the other three algorithms are analyzed based on the results of conformal UCA composed of directional antennas, integrated simulation and anechoic chamber experiments.

### 4.1. Computer Simulation Experiments

In the section of simulation experiments, a conformal UCA composed of 8 directional antennas is simulated. The main parameters in the simulation system are shown in Table 2.

For each directional antenna, the simulated antennas pattern function g(θ) is:

(22) $$\{\begin{array}{l} g_{0}(\theta )=\frac{1}{2}+\sin c(\theta )\\ g_{m}(\theta )=g_{0}(\theta -\gamma_{m})\;m=1,2,\cdots ,M-1 \end{array}$$

For directional antennas in the array, the directional pattern has the same shape but the main beam is pointed differently. The main beam direction of each antenna is its normal direction and the angle interval of each antenna is the same, which is 45°.

For the multi-target scenario, same with “Signal Model” Section, it is assumed that the amplitudes of the echo signals for different targets is the same before being weighted by the antenna pattern, and all targets in the far-field are incoherent. At this time, not only the range parameters but also the angle parameters are different for two targets. The position parameters of the multi-target are clearly shown in Table 3 for the simulation experiments. The range and angle parameters of any two targets are different. In the Computer Simulation Experiments Section, S-MUSIC and the two-stage fast DOA estimation algorithm need to divide the array into sub-arrays, and the setting of the angle parameters of four targets is closely related to the sub-array division method. Among them, the angle of target 1 is near the edge of a certain sub-array FOV, and both the angle of target 2 and 3 within a certain sub-array FOV are relatively close, while the angle of target 4 is near the center of certain sub-array FOV. Based on the simulation setting, it is possible to comprehensively verify the performance of algorithms under different angle situations.

**Figure 4:**
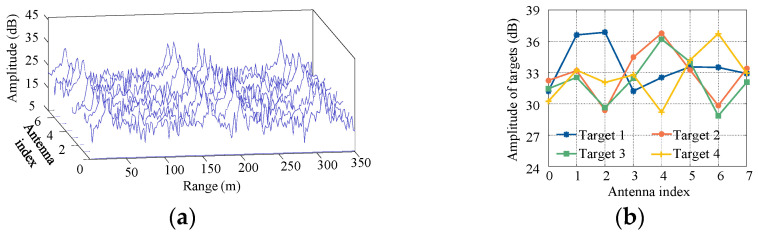
Amplitude of targets. (**a**) range-array 2D spectrum: (**b**) Target amplitudes in single range bin.

In simulation experiments, when SNR=0 dB, the targets’ amplitude information on each antenna is shown in Figure 4. From the range-array 2D spectrum (Figure 4a) and targets’ amplitudes in single range bin (Figure 4b), for a single target, the directivity of the directional antennas will lead to obvious differences in amplitude of each antenna. For target 1, the maximum amplitude difference on different antennas is 5.68 dB, and the amplitude differences of the other three targets (target 2~4) on different antennas is also significantly distinct (the corresponding maximum amplitude differences aresd 6.55 dB, 6.27 dB and 7.45 dB, respectively, Figure 4b).

#### 4.1.1. Spatial Spectrum Simulation

In single spatial spectrum simulation with SNR=0 dB, the estimation values, estimation errors and elapsed time of the two-stage fast DOA estimation algorithm and the other three algorithms (DA-MUSIC, S-MUSIC and Capon-like) are compared and analyzed. In order to evaluate the estimation performance, the DOA estimation error for the kth target is defined as $\Delta \theta_{k}=\left|\theta_{k}-{\hat{\theta }}_{k}\right|$, where ${\hat{\theta }}_{k}$ is the angle estimation value for the kth target.

**Figure 5:**
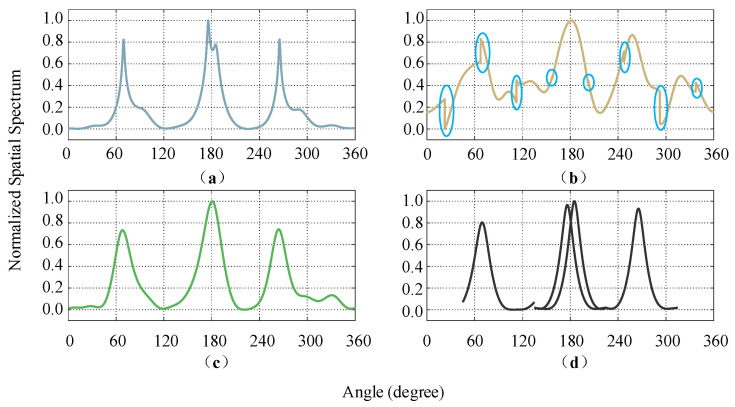
Spatial spectrum for different algorithms: (**a**) DA-MUSIC, (**b**) S-MUSIC, (**c**) Capon-like, and (**d**) two-stage fast DOA estimation.

The simulation spatial spectrum of different algorithms is shown in Figure 5. For the proposed two-stage fast DOA estimation and DA-MUSIC algorithm, four obvious spectrum peaks can be identified, which is the same as the targets number K set in the simulation setup (Figure 5a,d). However, for the S-MUSIC (Figure 5b) and the Capon-like (Figure 5c) algorithms, there is only one spectrum peak around 180.0°, so three obvious spectrum peaks are identified, which is less than the targets number K set in the simulation setup. In addition, due to the different noise subspaces corresponding to each sub-array, there are some breakpoints in the spatial spectrum for the S-MUSIC algorithm (blue ellipses in Figure 5b, and the angle position for these breakpoints is $\Psi =\{\psi_{m}|22.5{{}^{\circ}}^{}+45{{}^{\circ}}^{}*\left(m-1\right),m=1,2,\cdots ,M\}$.

The DOA estimation values and corresponding errors are shown in Table 4. For targets 1 and 4, compared to the estimation errors of DA-MUSIC, S-MUSIC and Capon-like algorithms ($0.3^{{}^{\circ}}$ and $0.0^{{}^{\circ}}$, $1.8^{{}^{\circ}}$ and $7.8^{{}^{\circ}}$, $1.7^{{}^{\circ}}$ and $1.6^{{}^{\circ}}$, respectively), the estimation errors of the two-stage fast DOA estimation algorithm are smaller ($0.1^{{}^{\circ}}$ and $0.0^{{}^{\circ}}$). For targets 2 and 3, the corresponding spectrum peaks mix together in the S-MUSIC and Capon-like algorithms and the peak angle positions after mixing are $181.4^{{}^{\circ}}$ and $180.0^{{}^{\circ}}$, respectively. Although the two-stage fast DOA estimation algorithm and the DA-MUSIC algorithm can well distinguish the two targets around $180.0^{{}^{\circ}}$, compared with the DA-MUSIC algorithm ($0.7^{{}^{\circ}}$ and $0.6^{{}^{\circ}}$), the DOA estimation errors of the two-stage fast DOA estimation algorithm are smaller ($0.3^{{}^{\circ}}$ and $0.0^{{}^{\circ}}$). In general, for the spatial spectrum experiment, compared with the other three algorithms (DA-MUSIC, S-MUSIC and Capon-like), the two-stage fast DOA estimation algorithm can effectively distinguish that multi-target and the corresponding estimation errors are the smallest.

The simulation experiments are conducted on the Window 10 PC platform with a i7-4810MQ CPU@2.8 GHz and 32 GB memory and the elapsed time in single estimation for four algorithms (DA-MUSIC, S-MUSIC, Capon-like and two-stage fast DOA estimation) can be seen in Table 5. In Table 5, the elapsed time shown for the different algorithms is the average time of 100 experiments. Compared to DA-MUSIC, S-MUSIC and Capon-like algorithms (0.391 s, 0.043 s and 0.432 s), the proposed two-stage fast DOA estimation algorithm takes the least time (0.035 s). For proposed algorithm, the elapsed time for each target is simulated and the corresponding elapsed time is shown in Table 6. Table 6 shows that the four elapsed times are approximated, and the elapsed time for different targets is not affected by the location of the target.

Combined with Table 1, it can be seen that the DA-MUSIC and the Capon-like algorithms need to calculate the covariance matrix $R_{xx}$ for array output data X and conduct EVD for $R_{xx}$. However, the computational complexity of calculating $R_{xx}$ and conducting EVD are $O\left(M^{2}L\right)$ and $O\left(M^{3}\right)$ respectively, so these two algorithms take a long time. S-MUSIC and the two-stage fast DOA estimation algorithms only need to calculate the covariance matrix $R_{xx}$ for the sub-array output data and conduct EVD for $R_{xx}$, so the elapsed time is less. In addition, the two-stage fast DOA estimation algorithms only need to search a spectrum peak in the FOV of a single sub-array, so the elapsed time is the least.

#### 4.1.2. Estimated Validity Simulation

According to the analysis of the above spatial spectrum simulation results (Figure 5), it is found that in the multi-target scenario, there is a phenomenon of spectrum peaks mixing among the similar targets in the angle dimension, which leads to the inability to effectively estimate target DOA. In the simulation experiment, the accuracy of the DOA estimation of four different algorithms (DA-MUSIC, S-MUSIC, Capon-like and two-stage fast DOA estimation) for each target is statistically analyzed to measure the effectiveness of each algorithm for DOA estimation of the multi-target; In a single experiment, if the estimation error of the kth target is $\Delta \theta_{k}\leq 3^{{}^{\circ}}$, it is considered that the estimation of the kth target in this experiment is successful. Under different conditions, 200 Monte Carlo experiments are conducted to define the estimation valid probability of kth target as $P_{d}^{k}$.

(23) $$P_{d}^{k}=\frac{N_{d}^{k}}{200}$$

where $N_{d}^{k}$ denotes the number of successful estimation trials in 200 Monte Carlo experiments. Here, a total of 7 different SNRs are set from −10 dB to 20 dB in step of 5 dB.

**Figure 6:**
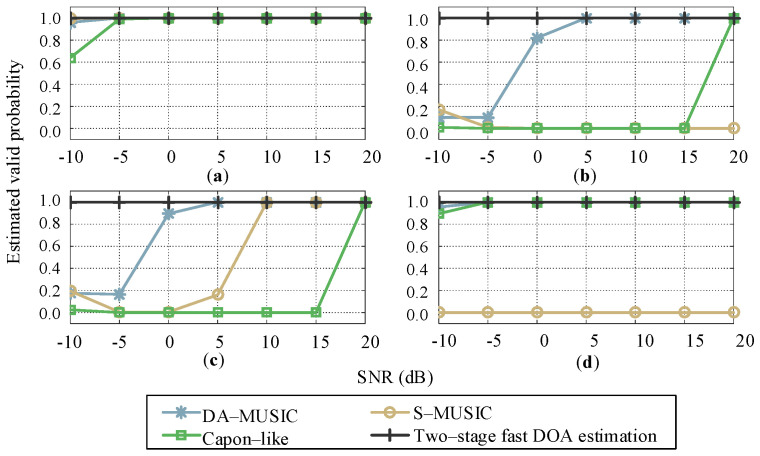
Estimated valid probability versus SNR for each target: (**a**) Target 1, (**b**) Target 2, (**c**) Target 3, and (**d**) Target 4.

From the estimated valid probability versus SNR for four targets (Figure 6), it can be seen that under different SNR situations, the proposed two-stage fast DOA estimation algorithm has an estimated valid probability of 1 for the four targets. In the pre-processing stage, since the two-stage fast DOA estimation algorithm decouples the information of multi-target by DFT, the estimated validity of each target is the same, which can realize the effective estimation of DOAs in the multi-target scenario. In addition, the estimation performance of the DA-MUSIC algorithm (the average estimated valid probability is 0.86) is better than that of the Capon-like algorithm (0.56), and the estimated validity increased with the increase of the SNR. However, the S-MUSIC algorithm has obviously different estimated valid probabilities for each target. Target 1 is located at the FOV edge of the sub-array, and a breakpoint leads to the spectrum peak, with high estimation performance (Figure 6a, when SNR≥−5 dB, the accuracy is 1). For targets 2 and 3, the estimation accuracy of target 2 is much lower than that of target 3 due to the mixing of the two close spectrum peaks (Figure 6b,c). For target 4, since the estimation error is bigger than $3^{{}^{\circ}}$, although it is located near the center of the FOV for the single sub-array, its estimation accuracy is 0 (Figure 6d).

#### 4.1.3. RMSE Statistical Analysis

In the multi-target scenario of the simulation experiment, 300 Monte Carlo experiments are conducted to calculate the corresponding root mean squared error (RMSE) to quantitatively analyze the DOA estimation accuracy for the four algorithms (DA-MUSIC, S-MUSIC, Capon-like and the two-stage fast DOA estimation). The variation range of the SNR is same as in the “Estimated validity simulation” Section and RMSE calculation formula is as follows:

(24) $$RMSE=\frac{1}{K}\sum_{k=1}^{K}\sqrt{\frac{1}{300}\sum_{Q=1}^{300}\left({\hat{\theta }}_{k(Q)}-\theta_{k(Q)}\right)}$$

where ${\hat{\theta }}_{k\left(Q\right)}$ represents the Qth estimation of $\theta_{k}$.

**Figure 7:**
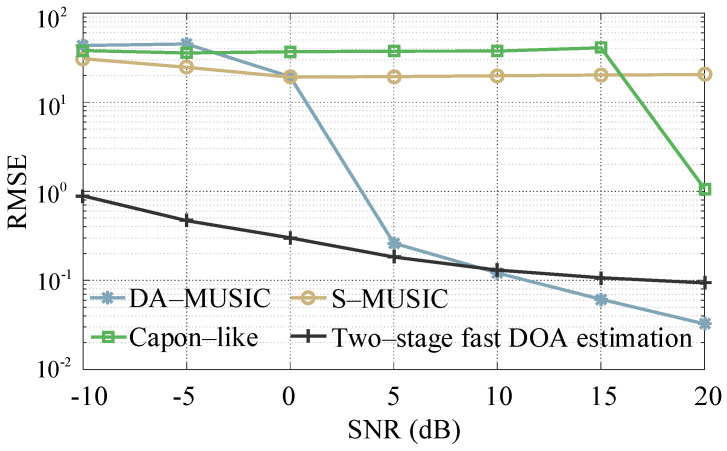
RMSE versus SNR.

The RMSE curves against the SNR for the four algorithms are shown in Figure 7. It can be seen that with the increase of SNR, the RMSE of the two-stage fast DOA estimation algorithm gradually decreases (from 0.89 to 0.09). For all the SNR situations in the simulation experiments, the RMSE of the S-MUSIC algorithm is relatively larger (the average RMSE is 22.07). Different from the S-MUSIC algorithm, the RMSE value of the Capon-like algorithm has an inflection point at SNR=15 dB (dropped from 40.85 to 1.07 hastily). As to the DA-MUSIC algorithm, the value of the RMSE within the range of simulated SNRs decreases from 43.37 to 0.03, and the infection point occurs when SNR=0 dB (RMSE decreased from 19.34 to 0.26). The reason for the existing inflection points of DA-MSUIC and the Capon-like algorithm is the close target 2 and 3 with close angles are distinguished. In summary, compared to the other three algorithms (DA-MUSIC, S-MUSIC and Capon-like), the proposed two-stage fast DOA estimation algorithm is robust in low SNR environments, which can estimate DOAs accurately for multi-target in low SNR environments.

### 4.2. Anechoic Chamber Experiments

**Figure 8:**
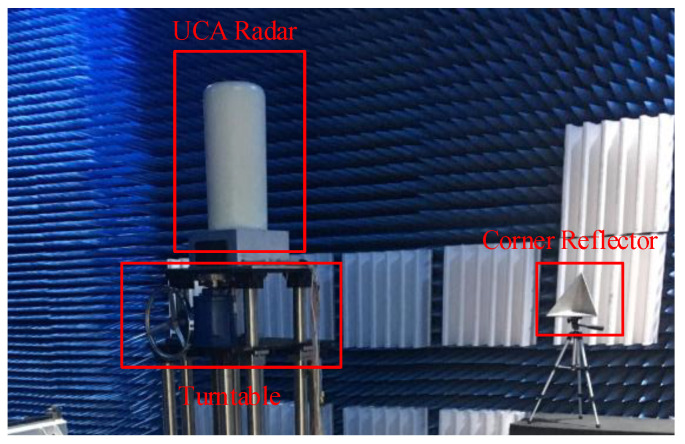
Experiment scenario in the anechoic chamber.

In order to further verify the performance of the proposed two-stage fast DOA estimation algorithm, an actual conformal UCA radar is used for the experiments in an anechoic chamber, and the experiment scenario is shown in Figure 8. In the anechoic chamber, the UCA radar is installed on the turntable, and a corner reflector is placed in the far-field. The range between the radar and the corner reflector is 4.7 m. The angle between radar and corner reflector is changed by the turntable to verify the DOA estimation performance of the two-stage fast DOA estimation algorithm under two different angle conditions. The angle between the UCA radar and the corner reflector in two experiments are $92.0^{{}^{\circ}}$ and $200.0^{{}^{\circ}}$, respectively.

**Figure 9:**
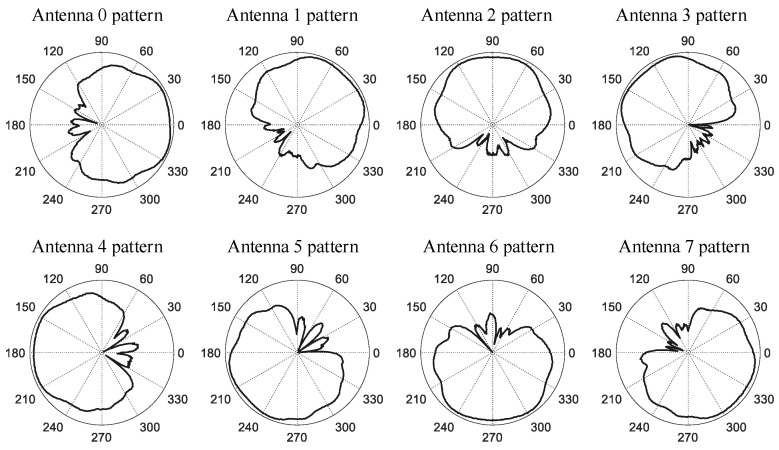
Antennas azimuth patterns.

The number of antennas in the actual UCA radar is M (M=8), and the shape of each antenna pattern is similar (Figure 9). However, the direction of the main beam for each antenna is its normal direction. The main system parameters of the actual UCA radar are shown in Table 7.

**Figure 10:**
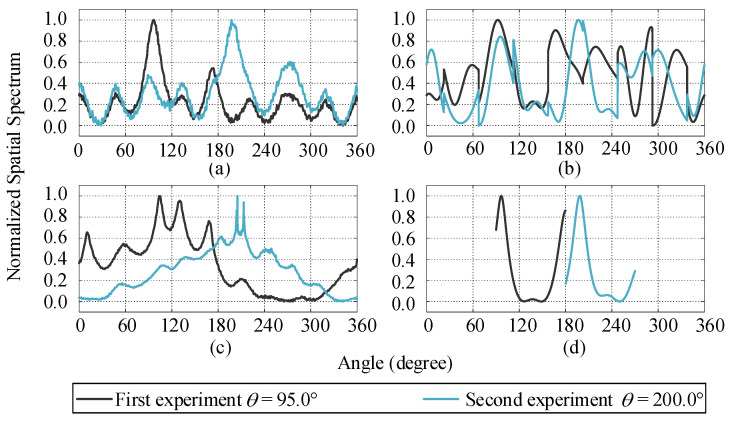
Four algorithms DOA spatial spectrum for two angle experiments (**a**) DA-MUSIC: (**b**) S-MUSIC: (**c**) Capon-like: (**d**) Two-stage fast DOA estimation.

The spatial spectrum of four different algorithms in two experiments is shown in Figure 10. For DA-MUSIC and Capon-like algorithms, due to the gain errors between the measurement and actual pattern, inevitably, there are a lot of burrs in the spatial spectrum (Figure 10a,c). In a single experiment, DA-MUSIC and the proposed two-stage fast DOA estimation algorithm have a single obvious peak in the spatial spectrum (Figure 10a,d). However, the spatial spectrum of the S-MUSIC and Capon-like algorithms are chaotic with a large number of obvious pseudo spectrum peaks (Figure 10b,c).

The DOA estimation values and corresponding errors in two angle experiments are shown in Table 8. In two different angle experiments, the estimation values of the proposed two-stage fast DOA estimation algorithm are $96.5^{{}^{\circ}}$ and $198.9^{{}^{\circ}}$, and the estimation values of the other three algorithms are $96.4^{{}^{\circ}}$ and $197.3^{{}^{\circ}}$, $91.9^{{}^{\circ}}$ and $197.0^{{}^{\circ}}$, $103.5^{{}^{\circ}}$ and $204.9^{{}^{\circ}}$, respectively. Compared to the other three algorithm, the estimation errors of the proposed two-stage fast DOA estimation algorithm are smaller, $1.5^{{}^{\circ}}$ and $1.1^{{}^{\circ}}$, respectively. Based on the result of Figure 10 and Table 8, it can be seen that the DOA estimation obtained by the two-stage fast DOA estimation algorithm is more accurate in the anechoic chamber experiments than for actual conformal UCA radar.

By comparing and analyzing the DOA estimation results of the DA-MUSIC, S-MUSIC, and Capon-like algorithms and the two-stage fast DOA estimation algorithm under the same experimental conditions of simulation and anechoic chamber, the below results are obtained: (1) for estimation accuracy, the two-stage fast DOA estimation has the smallest estimation error and a relatively smaller RMSE of 0.31 (the other three algorithms have errors of 15.46, 22.07 and 32.50, respectively); (2) for estimation validity, by analyzing the results of simulation experiments, it is found that the two-stage fast DOA estimation algorithm has a higher estimated valid probability of 1 (the other three algorithms have probabilities of 0.86, 0.38 and 0.56, respectively); and (3) for computational complexity, the elapsed time of the two-stage fast DOA estimation algorithms is 0.035 s in single estimation experiment (the other three algorithms are 0.391 s, 0.043 s and 0.432 s).

## 5. Conclusions

The two-stage fast DOA estimation algorithm is proposed based on a conformal UCA radar composed of directional antennas. For directional antennas, both phase and amplitude domain information on each antenna are used for DOA estimation. The proposed two-stage fast DOA estimation algorithm makes full use of this information to implement estimation with different precisions through different domain information. Rough DOA estimation is realized by using the amplitude information to divide sub-arrays and select sub-arrays. On the basis of the DOA rough estimation and based on the phase information of the single sub-array, the MUSIC algorithm is used to search for the spectrum peak in a single sub-array FOV to realize DOA precise estimation.

Aiming at the DOA estimation on the array radar composed of directional antennas, the simulation and anechoic chamber actual radar experiments prove that our research provides a useful reference for theoretical research and practical application, and higher estimation accuracy and lower elapsed time make it have a broad application prospect in the practical engineering field.

However, through theory analysis, it is found that the final DOA estimation value is vulnerable to the previous process result for the proposed two-stage fast DOA estimation algorithm. For the DOA estimation, the result of target detection will affect the subsequent rough-precise stage. In addition to this, when two targets are in the same range of bin index, the accuracy of target recognition will be affected if the array slice processing results are not satisfactory.

In the future, we will focus on solving the estimation loss efficacy for the two-stage fast DOA estimation when the targets are in the same range. In addition, an excellent target detection algorithm is another key work.

## Figures and Tables

**Table 1:** Computational complexity analysis for four algorithms.

Algorithm Name	Computational Complexity
DA-MUSIC	$O(M^{2}L+M^{3}+\frac{2\pi }{\kappa }\left(M+2M(M-K)+4(M-K))\right)$
S-MUSIC	$O\left(N_{s}{M_{s}}^{2}L+2N_{s}{M_{s}}^{3}+\frac{2\pi }{\kappa }\left(2{M_{s}}^{2}+4M_{s}\right)\right)$
Capon-like	$O\left(M^{2}L+M^{3}+M+\frac{4\pi }{\kappa }M^{2}\right)$
Two-stage fast estimation	$O\left({M_{q}}^{2}+{M_{q}}^{3}+\frac{2\pi }{M\kappa }K\left(2M_{q}\left(M_{q}-1\right)+4\left(M_{q}-1\right)\right)\right)$

**Table 2:** Radar parameters in simulation experiments.

Parameters Name	Notations	Values
Starting frequency	$f_{0}$	9 GHz
Bandwidth	B	100 MHz
Antenna number	M	8
Antenna 3 dB beam width	$\theta_{0.5}$	114.8°
Array radius	r	λ
Snapshots	L	4096

**Table 3:** Multi-target position parameters.

Target	$R_{k}\;\left(m\right)$	$\theta_{k}\;\left({}^{\circ}\right)$
1	200.0	69.3
2	308.3	176.2
3	24.8	185.4
4	160.0	265.8

**Table 4:** Multi-target DOA estimation values and errors for the four different targets.

**Estimation and Error**	**DA-MUSIC**	S-MUSIC	Capon-Like	Two-Stage Fast DOA Estimation
$V_{1}$	$69.0^{{}^{\circ}}$	$67.5^{{}^{\circ}}$	$67.6^{{}^{\circ}}$	$69.4^{{}^{\circ}}$
$V_{2}$	$176.9^{{}^{\circ}}$	$181.4^{{}^{\circ}}$	$180.5^{{}^{\circ}}$	$176.5^{{}^{\circ}}$
$V_{3}$	$184.8^{{}^{\circ}}$			$185.4^{{}^{\circ}}$
$V_{4}$	$265.8^{{}^{\circ}}$	$258.0^{{}^{\circ}}$	$264.2^{{}^{\circ}}$	$265.8^{{}^{\circ}}$
$\Delta \theta_{1}$	$0.3^{{}^{\circ}}$	$1.8^{{}^{\circ}}$	$1.7^{{}^{\circ}}$	$0.1^{{}^{\circ}}$
$\Delta \theta_{2}$	$0.7^{{}^{\circ}}$	$5.2^{{}^{\circ}}$	$4.3^{{}^{\circ}}$	$0.3^{{}^{\circ}}$
$\Delta \theta_{3}$	$0.6^{{}^{\circ}}$	$4.0^{{}^{\circ}}$	$4.9^{{}^{\circ}}$	$0.0^{{}^{\circ}}$
$\Delta \theta_{4}$	$0.0^{{}^{\circ}}$	$7.8^{{}^{\circ}}$	$1.6^{{}^{\circ}}$	$0.0^{{}^{\circ}}$

Note. $V_{1}\sim V_{4}$ are the estimation values for targets 1~4, $\Delta \theta_{1}\sim \Delta \theta_{4}$ are the corresponding estimation errors for targets 1~4.

**Table 5:** Elapsed time for four algorithms.

Algorithm	DA-MUSIC	S-MUSIC	Capon-Like	Two-Stage Fast DOA Estimation
Elapsed time (s)	0.391	0.043	0.432	0.035

**Table 6:** Elapsed time of the two-stage fast DOA estimation algorithm for four targets.

Target	1	2	3	4
Elapsed time (ms)	8.779	8.582	9.008	8.884

**Table 7:** Actual UCA radar parameters in the anechoic chamber experiments.

Parameters Name	Notations	Values
Starting frequency	$f_{0}$	9.3 GHz
Bandwidth	B	400 MHz
Antenna number	M	8
Antenna 3 dB beam width	$\theta_{0.5}$	$92.0^{{}^{\circ}}$
Array radius	r	λ

**Table 8:** Anechoic chamber experiments values and errors.

Estimation and Error	DA-MUSIC	S-MUSIC	Capon-Like	Two-Stage Fast DOA Estimation
Angle 1	$96.4^{{}^{\circ}}$	$91.9^{{}^{\circ}}$	$103.5^{{}^{\circ}}$	$96.5^{{}^{\circ}}$
Angle 2	$197.3^{{}^{\circ}}$	$197.0^{{}^{\circ}}$	$204.9^{{}^{\circ}}$	$198.9^{{}^{\circ}}$
$\Delta \theta_{1}$	$1.4^{{}^{\circ}}$	$3.1^{{}^{\circ}}$	$8.5^{{}^{\circ}}$	$1.5^{{}^{\circ}}$
$\Delta \theta_{2}$	$2.7^{{}^{\circ}}$	$3.0^{{}^{\circ}}$	$4.9^{{}^{\circ}}$	$1.1^{{}^{\circ}}$

Note. Angle 1 and angle 2 are the estimation values for the two different angle experiments; $\Delta \theta_{1}$ and $\Delta \theta_{2}$ are the corresponding errors.

## Data Availability

The data presented in this study are available on request from the corresponding author. The data are not publicly available due to privacy.
